# The Week's Work

**Published:** 1910-08-13

**Authors:** 


### THE WEEK'S WORK.

### TINKERING AND THOROUGHNESS.

One of the saddest reflections a hospital worker has to face is the thought that to a large extent his institution is merely a repairing shop, and that the good work that is done in it—work that often means an amount of personal service and self-sacrifice of which the public has but little conception, and that is often carried on under great difficulties—is largely negatived later on by the unhealthy environment in which "repaired" patients manage to exist. Saddest of all is the knowledge that where an institution is poor and struggling, where every penny must go towards defraying internal expenses, this method of repair must be seriously hampered owing to the want of funds to provide for the patients during convalescence. It is easy enough to lay down the fundamental rule that every hospital should have attached to it a convalescent home and an up-to-date social service department whose object should be to trace out old patients. In theory such a rule is all very well. In practice, however, who is likely to uphold it, especially at a time when already the notion is getting abroad that hospitals are unduly extravagant and ought to have their finances more closely looked into? We need not even discuss the astonishment of the average "man in the street" when such a proposition is expounded to him; after all, it is not his business to be familiar with the faults of the present system, under which a tuberculous child is admitted to a hospital for a couple of months and then turned out to go home and live the same insanitary life it lived before, with the inevitable result that in less than a year's time it has another bed in the repairing shop. But how many hospital workers are there who would be willing to agree that thoroughness in this respect is imperatively necessary? We are all at one that thoroughness is necessary enough in other matters that concern a hospital, but on this point of an extension of social work it is probable that no two of us would be in solid agreement.

### THE CASE FOR THOROUGHNESS.

A Continental hospital authority, whose opinion is entitled to consideration by reason of the fact that it is founded upon an experience that extends back into the 'fifties, has argued that, so far as the patients are concerned, thoroughness implies not only good hospital treatment, but effective home care, and at least prolonged convalescent home treatment. On this basis every hospital ought to maintain a convalescent home entirely out of proportion to the number of beds or the total number of in-patients treated every year, the reason for this disproportion being the fact that the home will receive a large number of out-patients as well. The out-patient "chronic," especially in the case of children, is often the product of insanitary surroundings, and the eternal tinkering that the physician or surgeon is forced to have recourse to will never be effective unless the patient can have the benefit of pure air, wholesome food, and skilful attention. Gradually we are coming to recognise the importance of such absolute thoroughness as this entails in the case of school children, for the establishment of school clinics is merely a matter of time. But we have not yet grasped the fact in its entirety so far as hospitals, and especially their out-patient departments, are concerned. We are not now arguing in favour of the retention of such departments, but the fact remains that so long as these departments are with us we shall have to take into account the conditions to which they give rise and the class of patient they bring. At present it is left largely to the personal interest of the staff to deal with questions of direct after-treatment beyond the walls of the institution. In the majority of cases such personal interest is, from the very nature of out-patient work, impossible. Where organised attempts have been made to deal with the problem by means of committees and sub-committees of the staff, the success which has attended their efforts has not been singularly brilliant. At a New York hospital there is, we believe, a staff committee the members of which visit every out-patient and attempt to improve the conditions under which such a patient lives, and so prevent the accumulation of extraneous factors which might cause a relapse. In many other hospitals there are special social service organisations, on which members of the medical staff have seats, and these organisations do excellent work; but they are handicapped through want of co-ordination and co-operation, and, above all, through the disinclination that exists among hospital workers to face the problem boldly and to admit that thoroughness is the only real motto that a hospital ought to possess. A dozen patients cured, and, moreover, prevented from relapsing, are far better than a hundred tinkered up and left exposed to the brunt of the same conditions of insanitation and want of hygiene which originally caused them to seek relief at the hospital gates.

### THE CASE AGAINST.

That is the case in favour of devoting a far larger proportion of the institutional funds to the maintenance of convalescent homes, outdoor camps, and health stations, and to schemes of social work than is at present allowed. Against it are to be set the arguments which the opponents of the more complete identification of the hospital with the objects of the home have to bring forward. In the first place they contend that it is not permissible to divert the funds of the hospital to outside purposes. If such purposes are desirable the obvious thing is to create special funds, such as, for instance, the already popular Samaritan Funds, to finance them. They maintain that social work must be left to outside agencies, not to the institutions themselves, and that all that is necessary is for a hospital to keep up a small convalescent home in which in-patients may be tended for a comparatively short period. The justification for this policy is found in the argument that a hospital exists to do the greatest amount of good to the greatest number of people in the shortest possible time. As hospitals exist to-day, as repairing shops mainly, this is doubtless true; but those who claim that they serve a higher and nobler object, that they have a national as much as a purely medical aspect, will scarcely agree that the best hospital is that institution which bundles out its patients in the shortest possible time to make room for new ones, regardless of the future of such dismissed patients. As a working rule the adoption of the "short standard" is doubtless reasonable enough, for when the average length of stay of a patient in a certain hospital rises to thirty or more days it may in general be taken that there is something seriously wrong with that institution. But the efficiency of a modern hospital cannot be judged exclusively by the average length of stay of patients in its wards. Where a usefully large convalescent home is attached to a hospital that average will be greatly lowered, and the tendency will be to lower it still more, and therefore to increase the serviceableness of the hospital by permitting it to deal with a larger number of patients, if the staff had reason to believe that the House Committees would look with favour upon a gradual rise in the expenses of the homes.

### OUTSIDE OR INSIDE ORGANISATION.

Nor is it always a convalescent home that is wanted. Many patients require the open air—God's glorious oxygen, as Gordon called it—reasonably pure food, and separation, for a time at least, from a sordid and unwholesome environment. Some arrangement for the establishment of hospital camps might well be thought out, on the plan now adopted, we believe, by certain charitable organisations here and abroad. The relative cost per patient of such camps would be far lower than the cost of convalescent homes, and their usefulness would be greater owing to the fact that they would be able to deal with a larger number of patients. The class of patient which they would receive, of course, is different from that which would be sent to the convalescent home, and in certain other respects they would materially differ from the homes—so much so, in fact, that we doubt whether hospital workers (and more especially those who have not had actual experience of out-patient cases) would feel inclined to regard them as coming within the scope of hospital politics at all. In any case no hospital, in London at least, appears to be in a position to establish such camps without calling in the help of outside organisations, and it is perhaps more desirable that some outside association should be formed for the purpose of dealing with such semi-social reform schemes than that an institution which is primarily there for the reception of sick people should run the risk of being popularly regarded as treating well people to a cheap holiday. But whether the social work—of which health camps are only a small portion—is to be arranged from inside the institution or whether it is to be delegated to outside societies, with the encouragement and moral support of the hospital, does not so much matter so long as hospital workers are fair and frank enough to admit that such work is imperatively necessary for an institution that wishes to maintain a reputation for thoroughness.

### THE DRUG MARKET.

Very little business has been done during the week, and no noticeable improvement is expected until the holiday season is over. The downward tendency in the price of opium has been checked, perhaps only temporarily, but prices for the time being are slightly higher than a week ago. There has been no further alteration in the price of codeine. Buchu leaves are again much dearer, and it would be difficult to buy even at a price 25 per cent. higher than was quoted a fortnight ago. Atropine is slightly lower in price. Acetic acid is dearer.

